# Dynamic Changes in Seed Germination under Low-Temperature Stress in Maize

**DOI:** 10.3390/ijms23105495

**Published:** 2022-05-14

**Authors:** Aiju Meng, Daxing Wen, Chunqing Zhang

**Affiliations:** State Key Laboratory of Crop Biology, Agronomy College, Shandong Agricultural University, Tai’an 271018, China; 18209466348@163.com (A.M.); dxwen@sdau.edu.cn (D.W.)

**Keywords:** transcriptome, low temperature, ribosome, peroxidase activity, phenylpropanoid biosynthesis pathway

## Abstract

Low-temperature stress delays seed germination in maize. Different maize inbred lines display various low-temperature resistance, but the dynamic changes in seed germination under low-temperature stress in maize remain unknown, especially at the transcriptome level. In this study, low-temperature-resistant maize (RM) inbred line 04Qun0522-1-1 had a significantly faster germination speed than low-temperature-sensitive maize (SM) line B283-1 under low-temperature stress. Moreover, the total antioxidant capacity, superoxide dismutase, and peroxidase activities were notably higher in the RM line than in the SM line from 3 to 6 d. In contrast, the SM line showed significantly higher malondialdehyde (MDA) content than the RM line at 6 d. Gene ontology (GO) enrichment analysis showed that in 2dvs0d, both SM and RM lines displayed the downregulation of ribosome-related genes. Moreover, photosystem II and heat shock protein binding-related genes were also downregulated in the SM line. In 4dvs2d, the RM line showed a higher degree of upregulation of the ribosome and peroxidase (POD)-related genes than the SM line. In 6dvs4d, POD-related genes were continuously upregulated in both SM and RM lines, but the degree of upregulation of the genes was higher in the SM line than in the RM line. Moreover, vitamin B6-related genes were specifically upregulated in the RM line. Kyoto Encyclopedia of Genes and Genomes (KEGG) enrichment analysis showed that in 6dvs4d, phenylpropanoid biosynthesis was the most significantly enriched pathway in both SM and RM lines. Moreover, phenylpropanoid biosynthesis was also enriched in the RM line in 4dvs2d. More than half of the differentially expressed genes (DEGs) in phenylpropanoid biosynthesis were peroxidase, and the DEGs were similar to the GO enrichment analysis. The results provide new insights into maize seed germination in response to low-temperature stress.

## 1. Introduction

Maize (*Zea mays* L.) has many exciting characteristics relevant to basic and applied research [1,2]. For example, its highly diverse genome may contribute to its dispersal from the origin area to regions with substantially different environmental conditions [2,3]. As a thermophilic crop, maize is vulnerable to cold damage in early spring, which inhibits seed germination and seedling growth. At the germination stage, resistance to low temperatures is essential for plants growing in a temperate region. Rapid germination and seedling growth under low-temperature stress could enable early sowing in spring. However, the molecular mechanism underlying low-temperature resistance or sensitivity during seed germination in maize remains unknown, in contrast to the available information about the effects of cold stress on seedlings or at the whole-plant level [4,5,6].

Seed germination is a complex process involving the following three stages: (i) the rapid water-absorption stage (i.e., imbibition stage); (ii) the lag period during the germination stage; and (iii) the accelerated embryo growth period during the germination stage [7]. The optimum temperature of seed germination is about 25 °C in maize, and temperatures below 15 °C may cause low-temperature stress [8,9]. There is almost a complete lack of development in maize exposed to temperatures below 10 °C [10]. Low-temperature stress decreases the seedling emergence rate and increases the chances of infection by pathogenic soil bacteria, seriously reducing the maize yield [11]. Therefore, the effects of low-temperature stress on maize seed germination need further study.

Under low-temperature stress, plants undergo a series of physiological and morphological changes. The physiological changes include cell membrane hardening, reactive oxygen species (ROS) accumulation, protein instability, and metabolic disorders [12]. The morphological changes include delayed germination, decreased germination rate, and inhibited seedling growth [13]. ROS are also considered a signal molecule in different organisms, which can affect various physiological processes in plants. To eliminate the toxicity of ROS, plants have established an excellent antioxidant defense system, including a large number of enzymatic antioxidants and nonenzymatic antioxidants. Some ROS scavengers can resist environmental stress by regulating the expression of antioxidant enzyme genes, such as superoxide dismutase (SOD), peroxidase (POD), and catalase (CAT) [14,15,16]. As a cofactor, Vitamin B6 (VB6) plays an essential role in the enzymatic reaction and is related to cellular oxidative stress defense [17].

Ribosomes convert the genetic information of messenger RNA into functional proteins [18]. As an essential, complex, and energy-consuming process, ribosome biosynthesis is accomplished by endogenous signals [19] and environment stimuli [20], such as ambient temperature [21,22]. Previous studies identified the processing pathway of precursor ribosomal RNA in rice and showed that ribosomal biogenesis was rapidly inhibited by low temperature [23]. 

Previous studies mainly focus on maize seedling responses to low temperatures [4,5]. The mechanism underlying maize seed germination under low-temperature stress is still ambiguous. The objective of this study was to investigate the dynamic changes in seed germination under low-temperature stress in maize, especially at the transcriptome level. We first detected the morphological and physiological changes in seed germination under low-temperature stress in two maize inbred lines with different low-temperature resistance. Subsequently, we analyzed the transcriptome changes of seeds germinated at low temperatures for 0, 2, 4, and 6 days. The results provide new insights into maize seed germination in response to low-temperature stress.

## 2. Results

### 2.1. Effects of Low-Temperature Stress on Seed Germination in Two Maize Inbred Lines

**Figure 1:**
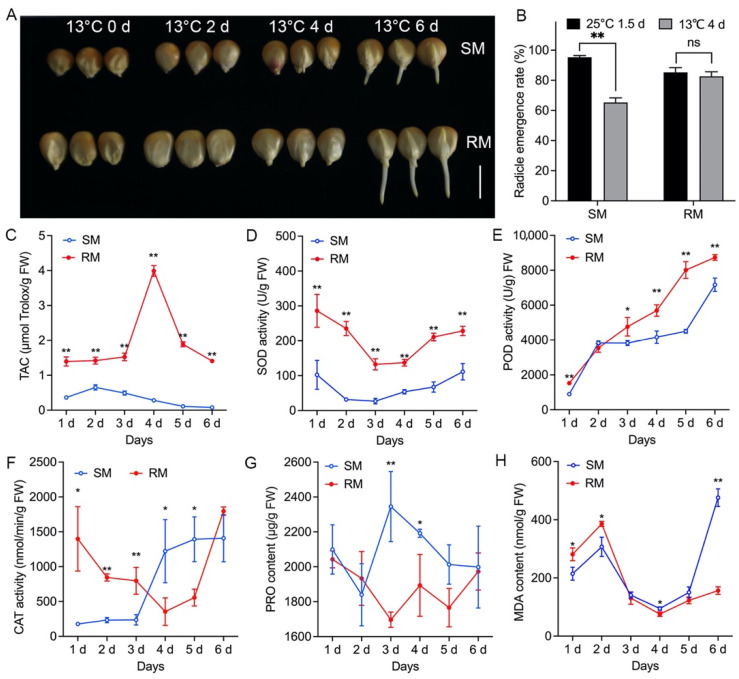
Germination characteristics, antioxidant activities, PRO content, and MDA content in two maize inbred lines under low-temperature stress. (**A**) Phenotypes of seed germination, (**B**) Percentage of radicle protrusion, (**C**) TAC, (**D**) SOD activity, (**E**) POD activity, (**F**) CAT activity, (**G**) PRO content, (**H**) MDA content in two maize inbred lines at 13 °C germinated from 1 to 6 d. Data are means ± standard deviation (*n* = 3 replications of 30 plants). Asterisks denote a significant difference according to an unpaired Student’s *t*-test (*: *p* < 0.05; **: *p* < 0.01; ns, not significant.

To investigate the effects of low-temperature stress on maize seed germination, we detected the differences in radicle emergence rate, antioxidant activities, proline (PRO) content, and lipid peroxidation, expressed as malondialdehyde (MDA) content, in the SM and RM lines. The two inbred lines displayed radicle protrusion at about 4 d under low-temperature stress (Figure 1A). Compared with the SM line, the radicle emergence rate of the RM line was higher at 13 °C for 4 d (Figure 1B), and the RM line had a longer radicle at 13 °C for 6 d (Figure 1A). Moreover, the radicle emergence rate in both the SM and RM lines was more than 80% when seeds germinated at 25 °C for 1.5 d (Figure 1B). In the RM line, there was no significant difference in the radicle emergence rate between seeds germinated at 25 °C for 1.5 d and 13 °C for 4 d. However, the SM line had a significantly lower radicle emergence rate at 13 °C for 4 d than at 25 °C for 1.5 d, indicating the SM line was more vulnerable to low-temperature stress. An antioxidant activities analysis showed that the RM line had significantly higher total antioxidant capacity (TAC) and SOD activities than the SM line from 1 to 6 d (Figure 1C,D). At 4 d, the RM line had extremely high TAC, which may be related to the high radicle emergence rate at that moment. Except at 2 d, the POD activities of the RM line were also significantly higher than the SM line (Figure 1E). The RM line had notably higher CAT activities from 1 to 3 d than the SM line, whereas the trends of CAT activities were opposite from 4 to 5 d (Figure 1F). There was no significant difference in PRO content between the SM and RM lines at most time points, except 3 and 4 d (Figure 1G). The MDA content was significantly higher in the RM line than in the SM line from 1 to 2 d, but the RM line had lower MDA content from 4 to 6 d (Figure 1H). Furthermore, the RM line had the lowest MDA content and the highest TAC at 4 d (Figure 1C,H). At 6 d, the SM line showed the highest MDA content, which may be because the SM line was more vulnerable to low-temperature stress after coleoptile protrusion from the seed coat.

### 2.2. Transcriptome Analysis of Seed Germination in Two Maize Inbred Lines under Low-Temperature Stress

**Figure 2:**
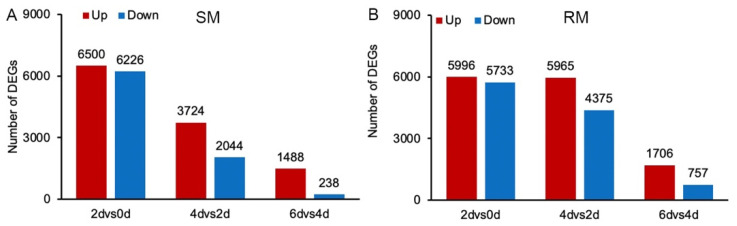
Number of DEGs in two maize inbred lines during low-temperature germination. (**A**) The number of up regulated and down regulated DEGs SM lines. (**B**) The number of up regulated and down regulated DEGs RM lines. SM: low-temperature sensitive maize inbred line; RM: low-temperature resistant maize inbred line.

To explore the dynamic changes in seed germination under low-temperature stress, we analyzed the transcriptomes in the SM and RM lines during seed germination under low-temperature stress. Three biological replicates were analyzed per time point for a total of 24 sequencing libraries. After removing adapters and sequences with low-quality regions, between 39.53 and 47.14 million clean reads remained for the three replicates of two lines at 0, 2, 4, and 6 d (Supplemental Table S1). From these, 33.42–41.56 million clean reads were mapped to the B73 maize reference genome using HISAT [24]. Finally, 78.39–87.10% of the reads were uniquely mapped to the reference genome sequence, whereas 2.46–3.78% were mapped to multiple reference genome sequences. To identify the differentially expressed genes (DEGs) in maize seeds germinated under low-temperature stress, we performed a pairwise differential expression profile analysis for all time points. The cutoffs were |log_2_ (fold-change)| ≥ 1 and adjusted *p* < 0.05. Based on the comparisons between consecutive time points, there were more DEGs in the SM line in the 2dvs0d comparison than those in the 4dvs2d and 6dvs4d comparisons (Figure 2A). In the RM line, there were more DEGs in the 2dvs0d and 4dvs2d comparisons than in the 6dvs4d comparison (Figure 2B). Moreover, there were more upregulated DEGs than downregulated DEGs for all comparisons (Figure 2).

### 2.3. Peroxidase Activity-Related Genes Are Involved in Response to Low-Temperature Stress during Seed Germination

**Figure 3:**
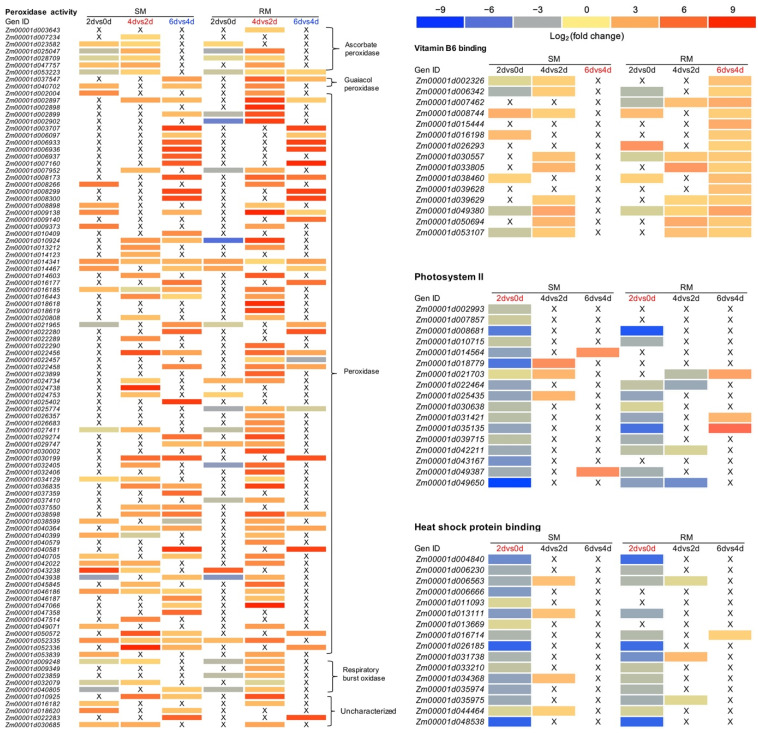
Heat map of selected DEGs enriched in GO terms. Detailed lists of the DEGs are shown in Supplementary Tables S3–S7. SM: low-temperature sensitive maize inbred line; RM: low-temperature resistant maize inbred line. The color code from blue to red suggests the expression level of the DEGs normalized as the log_2_ (fold change).

To gain insights into the functions of these DEGs, we performed a Gene Ontology (GO) enrichment analysis in three comparisons of 2dvs0d, 4dvs2d, and 6dvs4d. For the upregulated DEGs in 2dvs0d, the most significantly enriched GO term was the hydrolase activity (acting on acid anhydrides, GO: 0016817) in the molecular function group in both SM and RM lines (Supplemental Table S2). In 4dvs2d, the most significantly enriched GO term was the hydrolase activity (hydrolyzing o-glycosyl compounds, GO: 0004553, *p* = 1.30 × 10^−13^) in the molecular function group in the SM line, and the structural constituent of the ribosome (GO: 0003735, *p* = 4.86 × 10^−23^) in the molecular function group in the RM line. Moreover, ribosome (GO: 0005840) was the most significantly enriched GO term in the cellular component group in both SM and RM lines (Supplemental Table S2). In 6dvs4d, the most significantly enriched GO term was the peroxidase activity (GO: 0004601, *p* = 5.66 × 10^−20^) in the molecular function group in the SM line, and the protein heterodimerization activity (GO: 0046982, *p* = 2.14 × 10^−12^) in the molecular function group in the RM line. Moreover, the peroxidase activity (GO: 0004601, *p* = 2.38 × 10^−4^) was also enriched in the RM line in 6dvs4d and in the SM and RM lines in 4dvs2d (Supplemental Table S2). In 2dvs0d, the peroxidase activity (GO: 0004601) was not enriched in the SM and RM lines. Thus, we further analyzed the DEGs in peroxidase activity (GO: 0004601). The RM line had more DEGs and a higher degree of upregulation of the DEGs than the SM line in 4dvs2d, which were opposite in 6dvs4d (Figure 3, Supplemental Table S3). The results showed that peroxidase activity-related genes played essential roles in the response to low-temperature stress during seed germination, and the RM line displayed the upregulation of peroxidase activity-related genes earlier than the SM line.

In our previous study, RM seeds germinated under low-temperature stress showed the upregulation of vitamin B6-related genes compared with seeds germinated under normal temperature [25]. In the present study, we found that vitamin B6 binding (GO: 0070279, *p* = 1.39 × 10^−3^) was a specifically enriched GO term in RM-6dvs4d (Supplemental Table S2). In 6dvs4d, the RM line had 15 upregulated vitamin B6 binding-related genes, whereas no upregulated vitamin B6 binding-related genes were in the SM line (Figure 3, Supplemental Table S4). Moreover, there were several upregulated vitamin B6 binding-related genes in both 2dvs0d and 4dvs2d, but vitamin B6 binding (GO: 0070279) was not a significantly enriched GO term in the two comparisons (Figure 3). Therefore, vitamin B6-related genes may respond to low-temperature stress after radicle protrusion.

### 2.4. Photosystem II (PSII) and Heat Shock Protein Binding-Related GO Terms in the SM Line Are More Vulnerable to Low-Temperature Stress at the Early Stage of Seed Germination

For the downregulated DEGs in 2dvs0d, the most significantly enriched GO terms were the photosynthetic membrane (GO: 0034357, *p* = 3.55 × 10^−7^), thylakoid (GO: 0009579, *p* = 3.55 × 10^−7^), and thylakoid part (GO: 0044436, *p* = 3.55 × 10^−7^) in the cellular component group in the SM line, and the three GO terms were also enriched in the RM line (Supplemental Table S5). Photosystem II (GO: 0009523, *p* = 6.46 × 10^−6^) was specifically enriched in the SM line. Moreover, heat shock protein binding (GO: 0031072, *p* = 4.80 × 10^−3^) was also specifically enriched in the SM line. However, ribosome (GO: 0005840, *p* = 6.73 × 10^−26^) was the most significantly enriched GO term in the cellular component group in the RM line (Supplemental Table S5). For the downregulated DEGs in the SM line, only one GO term was enriched in 4dvs2d, and no GO term was enriched in 6dvs4d. Although twenty-four and five GO terms were enriched in 4dvs2d and 6dvs4d, respectively, in the RM line, the p values of the most significantly enriched GO terms in the two comparisons were higher than those in other comparisons (Supplemental Table S5). Thus, we further analyzed the DEGs involved in photosystem II, heat shock protein binding, and ribosome. In 2dvs0d, there were seventeen and eleven downregulated photosystem II in the SM and RM lines, respectively (Figure 3, Supplemental Table S6). Moreover, most genes showed a higher degree of downregulation in the SM line than in the RM line. Heat shock protein binding-related genes showed similar trends to photosystem II-related genes (Figure 3, Supplemental Table S7).

### 2.5. Low-Temperature Stress May Delay Seed Germination by the Downregulation of Ribosome-Related Genes

Numerous proteins are synthesized during seed germination and seedling establishment, which need ribosomes. Compared with the SM line, the RM line had more downregulated ribosome-related genes and a higher degree of downregulation of the genes in 2dvs0d, but the RM line had more upregulated ribosome-related genes and a higher degree of upregulation of the genes in 4dvs2d (Supplemental Table S8). In 6dvs4d, there were only three and four ribosome-related DEGs in the SM line and RM line, respectively. Therefore, both SM and RM lines showed the downregulation of ribosome-related genes at the early stage of seed germination under low-temperature stress, whereas the RM line converted to upregulate ribosome-related genes faster than the SM line before radicle protrusion (Supplemental Table S8). Moreover, radicle protrusion could be observed when the maize seeds germinated at 13 °C for 4 d or 25 °C for 1.5 d in both SM and RM lines. We analyzed the transcriptomes of seeds germinated at 13 °C for 4 d compared to seeds germinated at 25 °C for 1.5 d (4d-LTvs1.5d-NT). Interestingly, there were 47 downregulated ribosome-related genes in the SM line, whereas the RM line had one downregulated and one upregulated ribosome-related gene (Supplemental Table S8). Therefore, slow germination in the SM line under low-temperature stress may also be implicated in the downregulation of ribosome-related genes. 

### 2.6. Kyoto Encyclopedia of Genes and Genomes (KEGG) Enrichment Analysis of the DEGs in Dynamic Changes in Seed Germination under Low-Temperature Stress

**Figure 4:**
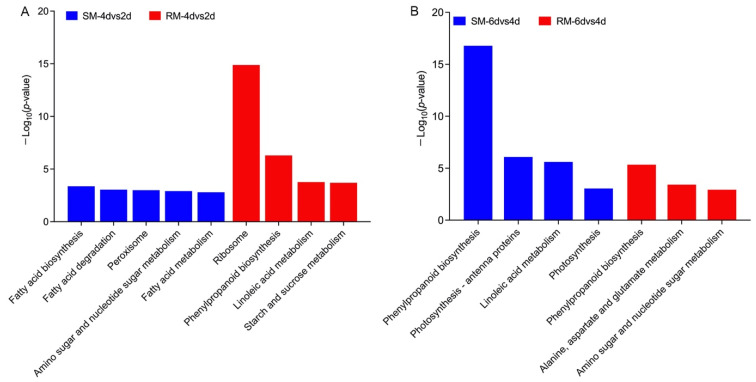
Significantly enriched KEGG pathways in (**A**) 4dvs2d and (**B**) 6dvs4d. SM: low-temperature sensitive maize inbred line; RM: low-temperature resistant maize inbred line; 4dvs2d: maize seeds germinated at 13 °C for 4 d compared with seeds germinated at 13 °C for 2 d; 6dvs4d: maize seeds germinated at 13 °C for 6 d compared with seeds germinated at 13 °C for 4 d.

To further clarify the metabolic pathways of the DEGs in the dynamic changes in seed germination under low-temperature stress, we performed a KEGG enrichment analysis. In 2dvs0d, there was no significantly enriched KEGG pathway in the SM line, and only the ribosome pathway was significantly enriched in the RM line (Supplemental Table S9). In 4dvs2d, there were five and four enriched pathways in the SM line and the RM line, respectively (Figure 4). The most significantly enriched pathway was ribosome, and the DEGs in the ribosome pathway were similar to the GO enrichment analysis. In 6dvs4d, the SM line had four enriched pathways, and the RM line had three enriched pathways. Phenylpropanoid biosynthesis was the most significantly enriched pathway in both SM and RM lines. Moreover, phenylpropanoid biosynthesis was also enriched in the RM line in 4dvs2d (Figure 4). Thus, we further analyzed the DEGs involved in phenylpropanoid biosynthesis. More than half of the DEGs were peroxidase, which were similar to the GO enrichment analysis (Supplemental Table S10). Moreover, the RM line had more upregulated genes involved in beta-glucosidase and phenylalanine ammonia lyase than the SM line in 4dvs2d.

### 2.7. Verification of the RNA-seq Data by Quantitative Real-Time Polymerase Chain Reaction (qRT-PCR) Analysis

**Figure 5:**
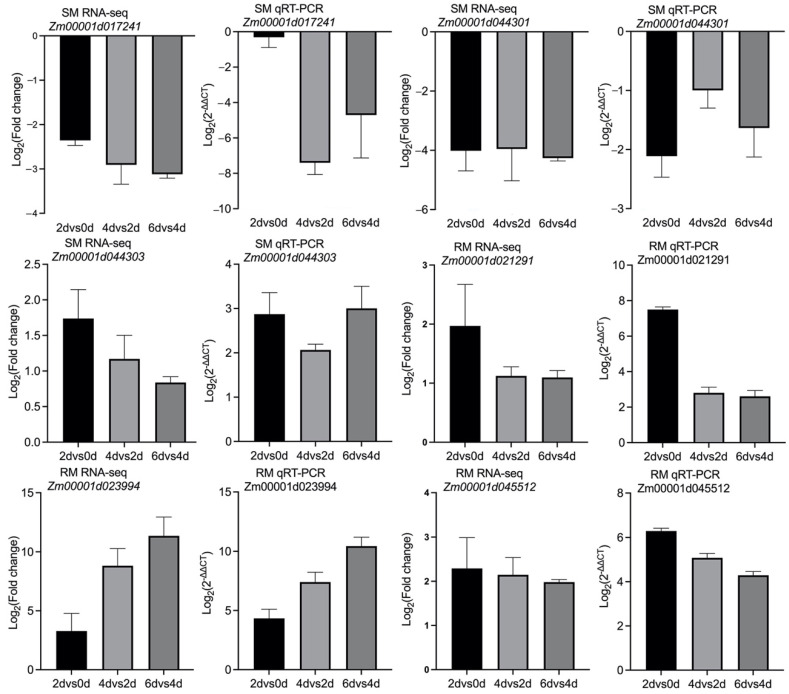
Validation of RNA-seq data by qRT-PCR. The qRT-PCR data were normalized against the internal reference gene data. The RNA-seq and qRT-PCR data are presented on the left and right, respectively.

To evaluate the RNA-seq data, genes encoding two-component response regulator-like APRR1 (*Zm00001d017241*), protein-serine/threonine phosphatase (*Zm00001d044301*), myosin 1 (*Zm00001d044303*), two-component response regulator-like APRR9 (*Zm00001d021291*), beta-glucosidase, chloroplastic (*Zm00001d023994*), and putative disease resistance RPP13-like protein 3 (*Zm00001d045512*) were randomly selected for qRT-PCR analyses. The results showed the similarity in the expression patterns for these genes determined by the RNA-seq and qRT-PCR analyses (Figure 5), which reflected the reliability of the RNA-seq data.

## 3. Discussion

The adverse effects of low-temperature stress are related to an imbalance in the oxidative system of plant cells [26]. Under low temperatures, an accumulation of ROS occurs [27], which may lead to excessive ROS that can cause various types of damage. For example, damage to proteins, lipids, and nucleic acids may lead to changes in the cell membrane fluidity of plant cells, as well as the inactivation of enzymes, inhibition of protein synthesis, and acceleration of macromolecules’ degradation [28]. Therefore, induced antioxidant defenses and limited ROS accumulation are necessary to restore redox homeostasis and normal metabolic activities under stress conditions. The efficient antioxidant system that restores redox homeostasis in plant cells consists of antioxidative enzymes (e.g., SOD and POD) and nonenzymatic components (e.g., mercaptan compounds, oxygen free radical scavengers, and other ROS inhibitors) [29]. In the present study, the POD activities in two inbred lines displayed a continuous upward trend during seed germination under low-temperature stress (Figure 1E). Moreover, the expression levels of the peroxidase-related genes enriched in the GO enrichment analysis showed similar trends with POD activities (Figure 3, Supplemental Table S2). The KEGG enrichment analysis showed that phenylpropanoid biosynthesis was the most significantly enriched pathway in the RM line in 4dvs2d and 6dvs4d. Interestingly, many DEGs in the pathway were annotated as peroxidase (Supplemental Table S10). Therefore, POD activity may play an important role in maize seed germination under low-temperature stress.

VB6 is an essential cofactor in the enzymatic reaction. Previous studies have proven that VB6 is an effective quencher of singlet oxygen and superoxide and has antioxidant activity [30,31]. In the present study, the GO enrichment analysis showed that vitamin B6 binding (GO:0070279, *p* = 1.39 × 10^−3^) was significantly enriched in RM-6dvs4d (Supplemental Table S2). Pyridoxal phosphate (PLP)-dependent transferase superfamily protein (*Zm00001d039629*), putative aminotransferase class III superfamily protein (*Zm00001d049380*), tyrosine aminotransferase homolog1 (*Zm00001d007462*), serine decarboxylase (*Zm00001d050694*), tyrosine aminotransferase (*Zm00001d053107*), glutamate decarboxylase (*Zm00001d033805*), and alanine aminotransferase 12 (*Zm00001d030557*) were all continuously upregulated only in the RM line in 4dvs2d and 6dvs4d (Figure 3, Supplemental Table S4). In 6dvs4d, VB6-related DEGs were significantly upregulated only in the RM line (Figure 3). Therefore, VB6 may play an important role in the response to low-temperature stress after radicle protrusion, which was present in the RM line and was not observed in the SM line.

The adaptation of photosynthetic organisms to low temperatures is considered to mainly be due to the increased fluidity of the cell membrane at low temperatures, which improves the ability of cells to repair damaged D1 protein [32]. PSII has always been considered to be the most sensitive process in photosynthesis [33,34]. Similar photosystem-related GO terms were significantly downregulated at 2dvs0d (Supplemental Table S5). In the present study, photosystem II (GO: 0009523, *p* = 6.46 × 10^−6^) was specifically enriched in the SM line for the downregulated DEGs in 2dvs0d. Moreover, the number of downregulated PSII-related genes was less in RM-2dvs0d than in SM-2dvs0d, and most genes showed a higher degree of downregulation in the SM line than in the RM line (Figure 3, Supplemental Table S6). Five genes (*Zm00001d014564*, *Zm00001d018779*, *Zm00001d007857*, *Zm00001d002993*, *Zm00001d021703*) implicated in oxygen-evolving enhancer protein were significantly downregulated only in the SM line (Figure 3, Supplemental Table S6). Therefore, the photosystem of the SM line was more vulnerable to damage at the early stage of seed germination under low-temperature stress, which may be related to the downregulation of genes encoding oxygen-evolving enhancer protein.

Heat shock proteins (HSP), as molecular chaperones, play essential roles in stress tolerance. In *Arabidopsis*, overexpression of *RcHSP17.8* enhanced SOD activity [35], whereas overexpression of *ZmHSP16.9* in tobacco increased the activities of POD, CAT, and SOD [36]. Overexpression of *GmHSP90A* in *Arabidopsis* reduced chlorophyll loss and lipid peroxidation [37]. In this study, heat shock protein binding (GO: 0031072, *p* = 4.80 × 10^−3^) was specifically enriched in the SM line for the downregulated DEGs in 2dvs0d. Moreover, the number of downregulated HSP-related genes was less in RM-2dvs0d than in SM-2dvs0d, and most genes showed a higher degree of downregulation in the SM line than in the RM line (Figure 3, Supplemental Table S7). Most HSP-related genes were annotated as DnaJ protein. DnaJ protein is an essential molecular chaperone in protein homeostasis and protein complex stability under pressure. Tomato chloroplast-targeted DnaJ protein is involved in maintaining PSII under low-temperature stress [38]. In this study, DnaJ 2 (*Zm00001d006666*) and chaperone protein DnaJ 3 (*Zm00001d013669*) were significantly downregulated in 2dvs0d only in the SM line. The other genes encoding DnaJ protein were significantly downregulated in both inbred lines, but the degree of downregulation in the RM line was less than that in the SM line (Figure 3, Supplemental Table S7). Interestingly, the gene expression patterns of PSII-related DEGs were similar to HSP-related DEGs (Figure 3). Therefore, the downregulation of PSII-related genes in 2dvs0d in the SM line may be related to the downregulation of HSP-related genes.

Ribosomal biogenesis is essential for plant growth and environmental adaptation [23]. In the present study, ribosome (GO:0005840) was significantly enriched in both SM and RM lines for downregulated DEGs at the early stage (2dvs0d) of seed germination under low-temperature stress. The number of ribosome-related downregulated genes in the RM line was more than that in the SM line. The RM line showed a higher degree of upregulation of the ribosome-related genes than the SM line before radicle protrusion (4dvs2d). Therefore, the RM line converted to upregulate ribosome-related genes faster than the SM line before the radicle protrusion (4dvs2d), and slow germination in the SM line under low-temperature stress may be related to the downregulation of ribosome-related genes. Previous studies have shown that eukaryotic ribosomes need to be synthesized to achieve long-term cold acclimation [39], similar to our results. 

**Figure 6:**
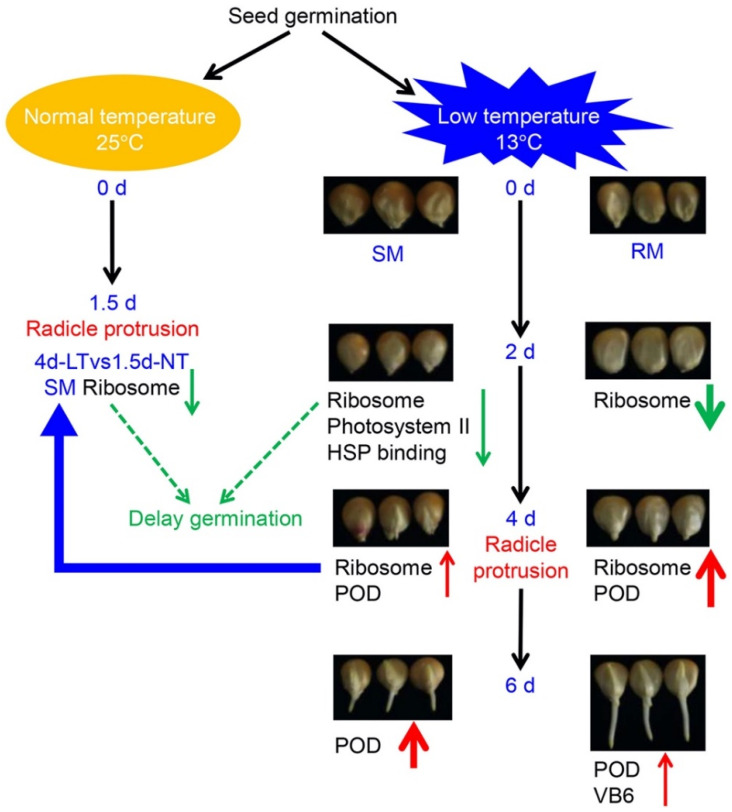
A possible network of the dynamic changes in seed germination under low-temperature stress in maize. The red and green arrows indicate upregulation and downregulation of genes, respectively. The thickened arrows indicate stronger changes induced by low-temperature stress. The blue arrow means transcriptome analysis of seeds germinated at 13 °C for 4 d compared to seeds germinated at 25 °C for 1.5 d (4d-LTvs1.5d-NT). The dotted lines indicate conjectural regulation.

Overall, we propose a model for the dynamic changes in seed germination under low-temperature stress in maize (Figure 6). Both SM and RM lines displayed the downregulation of ribosome-related genes at the early stage (2dvs0d) of seed germination under low-temperature stress. Moreover, photosystem II and HSP binding-related genes were also downregulated in the SM line. The RM line showed a higher degree of upregulation of the ribosome- and POD-related genes than the SM line before radicle protrusion (4dvs2d). Compared with maize seeds germinated at 25 °C for 1.5 d in the SM line, ribosome-related genes were also downregulated in seeds germinated at 13 °C for 4 d. Thus, low-temperature stress may delay seed germination by the downregulation of ribosome-related genes. After radicle protrusion (6dvs4d), POD-related genes were continuously upregulated in both SM and RM lines, but the degree of upregulation of the genes was higher in the SM line than in the RM line. Moreover, vitamin B6-related genes were specifically upregulated in the RM line.

## 4. Materials and Methods 

### 4.1. Plant Materials

Two maize inbred lines, SM and RM, were grown at the experimental station of Shandong Agricultural University, Shandong, China (approximately 117°90′ E longitude and 36°90′ N latitude) in June 2019. Considering the influence of seed maturity on germination, seeds were harvested at 50 days after pollination.

The seeds were germinated on a sprouting bed. Each germination box contained a 4-cm layer of silica sand with a 60% saturated moisture content. Thirty randomly selected maize seeds were sown in each germination box. The seeds were covered with a 2-cm layer of silica sand with a 60% saturated moisture content [40]. Three biological replicates were prepared. Several germination boxes were placed in a growth chamber at 25 ℃ for 1.5 d, and some germination boxes were placed in a growth chamber at 13 ℃ for 2, 4, and 6 days. The germination boxes were placed in a growth chamber at 13 °C for 4 d or at 25 ℃ for 1.5 d to detect the radicle emergence rate. All tissues were collected from the SM and RM seeds germinated at 13 ℃ for 0, 1, 2, 3, 4, 5, and 6 d or at 25 ℃ for 1.5 d used for later experiments. Thirty germinated seeds were pooled together for each sampling.

### 4.2. Determination of Antioxidant Enzyme Activities, PRO Content, and Lipid Peroxidation

All tissues of seeds germinated at 13 °C for 1 to 6 d were collected for enzyme activities, PRO content, and MDA content determination. Thirty germinated seeds were pooled together for each sampling, and the samples were then ground with liquid nitrogen. Each treatment included three biological replicates.

#### 4.2.1. Measurement of TAC

TAC was measured by 2,2′-azino-bis (3-ethylbenzothiazoline-6-sulfonic acid) diammonium salt (ABTS) [41], using the TAC Assay Kit (Comin, Suzhou, China) according to the manufacturer’s protocols. The mixed samples (0.1 g) were added to 1 mL extraction buffer and then centrifuged at 10,000× *g* for 10 min at 4 °C. The supernatant (10 μL) was added to the working solution (190 μL) and then mixed with the solution. The absorbance of the mixture was measured at 734 nm. Trolox was used for making a standard curve. The results were expressed as l μmol Trolox of TAC per 1 g of fresh weight (FW).

#### 4.2.2. Measurement of SOD Activity

SOD are metalloproteins catalyzing the dismutation of the superoxide free radical O_2_·^−^ to molecular oxygen and H_2_O_2_, according to Giannopolitis and Ries [42]. SOD was extracted using the SOD determination kit (Comin, Suzhou, China) according to the manufacturer’s protocols. The mixed sample (0.1 g) was added to 1 mL extraction buffer and then centrifuged at 8000× *g* for 10 min at 4 °C. Reagent 1 (45 μL), reagent 2 (2 μL), supernatant (18 μL), reagent 3 (35 μL), and reagent 4 (100 μL) were added in a 1.5 mL centrifuge tube. The solution was mixed and then stood at room temperature for 30 min. The absorbance of the mixture was measured at 560 nm. The results were expressed as l U of SOD activity per 1 g of FW.

#### 4.2.3. Measurement of POD Activity

POD catalyzes H_2_O_2_ oxidation of specific substrates and has characteristic light absorption at 470 nm [43]. POD was extracted using the POD determination kit (Comin, Suzhou, China) according to the manufacturer’s protocols. Reagent 1, reagent 2, and reagent 3 were mixed in a ratio of 2.6 (mL):1.5 (μL):1 (μL) to make the working solution. The mixed sample (0.1 g) was added to 1 mL extraction buffer and then centrifuged at 8000× *g* for 10 min at 4 °C. The supernatant (10 μL) was added to the working solution (190 μL) and then mixed with the solution. The absorbance of the mixture was measured at 470 nm. The fresh weight of each 1 g of tissue changes by 0.005 per minute in the A470 per mL reaction system as an enzyme activity unit.

#### 4.2.4. Measurement of CAT Activity

H_2_O_2_ has an absorption peak of 240 nm. CAT decomposes H_2_O_2_ so that the absorbance of the reaction solution at 240 nm decreases with the reaction time [44]. The CAT activity can be calculated according to the change rate of absorbance using the CAT Assay Kit (Comin, Suzhou, China) following the manufacturer’s protocols. The mixed sample (0.1 g) was added to 1 mL extraction buffer and then centrifuged at 8000× *g* for 10 min at 4 °C. The supernatant (35 μL) was added to the working solution (1 mL) and then mixed with the solution. The initial absorbance at 240 nm and the absorbance after 1 min were measured immediately at room temperature. The catalytic degradation of 1 nmol per g of tissue FW per minute is defined as one enzyme activity unit.

#### 4.2.5. Measurement of PRO Content

PRO was extracted with sulfosalicylic acid [45] using the PRO Assay Kit (Comin, Suzhou, China) following the manufacturer’s protocols. The mixed sample (0.1 g) was added to 1 mL extraction buffer and incubated at 95 °C for 10 min. The homogenate was centrifuged at 10,000× *g* for 10 min at 25 °C. The supernatant (250 μL) was transferred to a centrifuge tube, and 250 μL glacial acetic acid and 250 μL reagent 2 were then added. The solution was incubated at 95 °C for 30 min and then cooled down to room temperature. An amount of 500 μL toluene was added and mixed with the solution. An amount of 200 μL upper-layer solution was taken and the absorbance was measured at 520 nm. The results were expressed as l g of proline per 1 g of FW.

#### 4.2.6. Determination of Lipid Peroxidation

Lipid peroxidation was assessed from the content of thiobarbituric acid reactive substances [46], determined using a commercial kit (Comin, Suzhou, China). The mixed sample (0.1 g) was added to 1 mL extraction buffer and then centrifuged at 8000× *g* for 10 min at 4 °C. Reagent 1 (900 μL), supernatant (90 μL), and reagent 2 (300 μL) were mixed. The solution was incubated at 95 °C for 40 min and then cooled down to room temperature. The solution was centrifuged at 3000× *g* for 10 min. An amount of 200 μL supernatant was taken and the absorbance was measured at 535 nm. The results are expressed in nmol/g FW. 

### 4.3. RNA-seq and Data Analysis

Seeds germinated at 13 °C for 0, 2, 4, and 6 days were used for RNA extraction. Thirty germinated seeds were pooled together for each sampling and then stored at −80 °C before the RNA extraction. The frozen tissue samples were ground into a powder using a ball mill. Subsequently, the sample (0.1 g) was used for total RNA extraction using a Plant Total RNA Purification Kit (Bioflux, Beijing, China). Three biological replicates were prepared per sample for the Illumina transcriptome sequencing analysis. The RNA-seq libraries were constructed and sequenced as previously described [47].

The quality of RNA-seq data was tested. The retained clean reads were mapped to the RefGen_V4 maize reference genome and gene sequences (http://www.gramene.org/) using HISAT2 [24]. To quantify gene expression levels, feature Counts (v1.5.0-p3) were used to determine the number of reads mapped to each gene. The fragments per kilobase of transcript per million mapped reads method was used to estimate gene expression levels in the 24 analyzed samples. The analysis of differential expression between two conditions/groups (three biological replicates per condition) was performed using the DESeq2 R package (1.16.1) [48]. The p values were adjusted according to the Benjamini and Hochberg method. The DEGs (i.e., adjusted *p* < 0.05 and |log_2_ (fold-change)| ≥ 1) were considered for further analyses.

The DEGs were functionally characterized using the GO database [49]. All DEGs were grouped into the three main GO categories (biological process, cellular component, and molecular function) according to their GO terms using a publicly available database (http://www.geneontology.org/). In this study, the GO term with corrected *padj* < 0.05 was used as the GO term with significant enrichment of DEGs. To further clarify the biological functions of the DEGs, the significantly enriched metabolic or signal transduction pathways were identified based on a KEGG pathway enrichment analysis [50]. Similarly, the KEGG pathways with corrected *padj* < 0.05 were assigned as significantly enriched pathways.

### 4.4. Verification of DEGs by a qRT-PCR Analysis

To validate the DEGs identified from the RNA-seq analysis, we re-extracted the RNA of seeds germinated at 13 °C for 0, 2, 4, and 6 d in two lines. The following six genes were randomly selected for a qRT-PCR assay: *Zm00001d017241*, *Zm00001d044301*, *Zm00001d044303*, *Zm00001d021291*, *Zm00001d023994*, and *Zm00001d045512*. About 800 ng total RNA was used to synthesize cDNA by using the PrimeScript RT reagent Kit (Takara, Dalian, China). Subsequently, cDNA was diluted to 100 μL with ddH2O. All the qRT-PCR reactions were performed on an ABI Stepone plus Real-Time PCR System (Applied Biosystems, Foster City, CA, USA) in a 20 μL reaction volume, containing 10 μL 2 × SYBR Premix ExTaq (TaKaRa), 0.4 μL ROX, 0.4 μL of 10 μM primers, 2 μL cDNA, and 6.8 μL ddH2O. The qRT-PCR analysis was performed using the ABI StepOne Plus Real-Time PCR System (Applied Biosystems, CA, USA) and the SYBR^®^ Green Realtime PCR Master Mix (Toyobo, Japan). The maize *Actin* gene (GenBank accession number: *Zm00001d010159*) was used as an internal reference control [51]. The primers for the six selected genes were designed using the Primer Premier software (version 6.0) (Supplemental Table S11). Three biological replicates were included in the qRT-PCR analysis. The generated data were analyzed according to the 2^−ΔΔC^^T^ method [52].

### 4.5. IBM SPSS Statistical Version 21.0 Software

All experiments were performed in triplicate. Data were presented as mean ± SE. All the tests were performed using SPSS Version 21.0 for Windows (SPSS, Chicago, IL, USA).

## 5. Conclusions

Under low-temperature stress, the RM line had higher oxidase activity than the SM line during seed germination. Transcriptome analysis revealed that peroxidase- and ribosome-related genes played essential roles in the response to low-temperature stress at the germination stage. The RM line had a higher degree of upregulation of the ribosome and peroxidase genes than the SM line before radicle protrusion. The results provide new insights into the dynamic changes in seed germination in maize under low-temperature stress.

## Data Availability

Not applicable.

## Supplementary Materials

The following supporting information can be downloaded at: https://www.mdpi.com/article/10.3390/ijms23105495/s1.

## Abbreviations

RM	low-temperature-resistant maize
SM	low-temperature-sensitive maize
MDA	Malondialdehyde
GO	Gene ontology
POD	peroxidase
KEGG	Kyoto Encyclopedia of Genes and Genomes
DEGs	differentially expressed genes
ROS	reactive oxygen species
SOD	superoxide dismutase
POD	peroxidase
CAT	catalase
GPX	glutathione peroxidase
VB6	vitamin B6
PRO	proline
TAC	total antioxidant capacity
qRT-PCR	quantitative real-time polymerase chain reaction
PSII	Photosystem II
HSP	Heat shock proteins
ABTS	2,2′-azino-bis (3-ethylbenzothiazoline-6-sulfonic acid) diammonium salt
